# Anthocyanin-Induced Alterations in Substrate Oxidation During Exercise: A Narrative Review

**DOI:** 10.3390/nu18183018

**Published:** 2026-09-15

**Authors:** Matthew D. Cook, James L. Bateman, Mark E. T. Willems

**Affiliations:** 1 School of Sport and Exercise Science, University of Worcester, Henwick Grove, Worcester WR2 6AJ, UK; james.bateman@worc.ac.uk; 2 School of Sport, Science and Engineering, University of Chichester, College Lane, Chichester PO19 6PE, UK; m.willems@chi.ac.uk

**Keywords:** anthocyanins, lipid metabolism, carbohydrate metabolism, exercise, polyphenols

## Abstract

The bioactive flavonoid compound anthocyanin that is present in berries and other pigmented fruits has received interest for effects on substrate oxidation during exercise. The databases MEDLINE (via PubMed) and SPORTDiscus (via EBSCOhost) were searched from 4 March to 5 August 2026 for trials examining the exercise-induced metabolic effects of anthocyanin-rich foods and supplements. Twenty-three studies were included in this narrative review with observations from blackcurrant, blueberry, tart cherry, blackberry and elderberry. After short-term supplementation (up to 14 days), extract from New Zealand grown blackcurrants provided the strongest evidence, with many studies reporting up to 30% increases in fat oxidation and typically ~5–12% decreases in carbohydrate oxidation during continuous moderate-intensity exercise (cycling, walking and running). The exercise-induced metabolic responses appear to be influenced by the baseline respiratory exchange ratio, body composition, duration of dosing, and environmental exercise conditions. The findings for effects from other anthocyanin-rich sources were few and less clear. New Zealand blackcurrant extract increases moderate-intensity exercise-induced fat oxidation without alteration of the total energy expenditure. Mechanistic evidence for blackcurrant-induced effects suggests altered intramuscular triglyceride use and glycogen availability. Information from other anthocyanin-rich sources is limited for now. It cannot be excluded, however, that the changes in exercise-induced substrate oxidation may be anthocyanin-type (and thus berry) dependent, but more studies are needed on other anthocyanin-rich non-blackcurrant sources. Future studies are required to confirm mechanisms. And studies with experimental design and methodology are needed to examine responsiveness and the factors that may play a role, such as training status, exercise modality, age, and habitual diet, to advance our understanding for the implications of anthocyanin-rich supplementation for sport and exercise nutrition.

## 1. Introduction

Polyphenols are the largest and most diverse class of secondary plant metabolites and are typically grouped into four main categories: phenolics, flavonoids, stilbenes, and lignans. The four categories are primarily based on the differences in intrinsic chemical structures [1]. The largest flavonoid subgroup is the anthocyanins composed of anthocyanidin aglycones (i.e., anthocyanidins), such as pelargonidin, cyanidin, delphinidin, peonidin, petunidin, and malvidin, conjugated to carbohydrate moieties [2]. The anthocyanins provide colour to berries and fruits [3] and protect the plant genetic material from abiotic challenges such as high ultraviolet radiation, cold temperatures and water stress [4].

Health effects by anthocyanin intake [5,6,7,8] are largely attributed to the antioxidant and anti-inflammatory properties of the anthocyanins and anthocyanin-induced metabolites. Such properties are of particular interest for athletic populations due to the potential to enhance exercise performance and support post-exercise recovery. For example, exercise can induce increases in oxidative stress, inflammation, and muscle damage because of elevated metabolic activity [9]. Consequently, foods and supplements rich in anthocyanins are becoming increasingly popular as ergogenic aids within sports and exercise nutrition aimed at mitigating the sometimes non-beneficial physiological responses and contributing to overall antioxidant capacity [10]. The growing interest has also driven the development of a range of commercial products [11].

One application of anthocyanins within sports and exercise nutrition has been the effects on exercise-induced substrate oxidation. A growing number of controlled in vivo studies in humans have examined these effects of different anthocyanin-rich foods and berries, and evidence from blackcurrant supplementation studies in a recent systematic review and meta-analysis [12] demonstrated altered exercise-induced substrate oxidation. However, the effects from other anthocyanin-rich sources such as cherry or blackberry were not included in the systematic review [12] and are less clear. The present narrative review aims to be comprehensive regarding inclusion of the scientific literature on the effects of anthocyanin-rich sources on exercise-induced substrate oxidation.

## 2. Substrate Oxidation During Exercise

During continuous moderate-intensity exercise, fat and carbohydrate metabolism provide most of the ATP, with a negligible contribution of protein. The relative contributions and absolute rates of fat and carbohydrate oxidation are largely determined by the exercise intensity [13,14], in addition to other factors such as exercise duration, training status, gender, dietary intake and environmental conditions [15]. From a clinical and health perspective, the greater ability for exercise-induced fat oxidation has been linked to enhanced insulin sensitivity, improved metabolic flexibility, and a more favourable metabolic risk profile [16,17,18]. Increased fat oxidation during exercise may also support body fat reduction, with Wang et al. [19] demonstrating that 10 weeks of training at intensities eliciting maximal fat oxidation in overweight (BMI > 25 kg/m^2^) middle-aged (45–59 years) women resulted in significant reductions in body mass, fat mass, and abdominal fat.

For substrate use during exercise, exercise performance and endurance capacity can be strongly associated with the rate of fat oxidation, particularly under competitive conditions where the carbohydrate stores are becoming lower. For example, higher rates of maximal fat oxidation determined during laboratory-based cycling exercise were correlated with the overall ultra-endurance performance during the Ironman-distance triathlon in men [20]. Because exercise-induced fat oxidation may be linked to athletic performance and health, there is interest among athletes and sports nutrition practitioners for dietary supplements that can enhance this process.

In 2011, Jeukendrup and Randell [21] reviewed the available evidence for multiple dietary supplements (e.g., caffeine, L-carnitine, forskolin, fucoxanthin, kelp, tea, chromium, conjugated linoleic acid, and taurine) and concluded that only green tea and caffeine had been shown to increase fat oxidation during exercise. However, recent studies on the effects of anthocyanin-rich foods and supplements have provided evidence that they can enhance exercise-induced fat oxidation.

## 3. Purpose and Study Inclusion

The purpose of the present narrative review is to examine the evidence for anthocyanin-rich foods and supplements on exercise-induced substrate oxidation in humans. The review draws on the literature identified through searches of MEDLINE (via PubMed) and SPORTDiscus (via EBSCOhost) using the terms “anthocyanins substrate utilisation exercise,” “anthocyanins fat oxidation exercise,” and “anthocyanins carbohydrate oxidation exercise”; they were searched from 4 March 2026 to 5 August 2026. A total of 119 results were returned from the literature searches (Medline 91, EBSCO 28), with 38 duplicates removed and 81 unique titles remaining. Titles and abstracts were screened separately by two authors (MC and JB), with 19 full-text further studies chosen due to the relevance to the aim of the review, with no disagreements on papers chosen as relevant. Four further papers were found from reference lists and selected when the study examined the effects of anthocyanin-rich berries and drupes on exercise-induced substrate oxidation. The review offers a narrative synthesis of twenty-three studies with single food or supplementation intake and focuses on key findings, the mechanistic interpretation, and the potential for future research directions.

To strengthen the interpretation of the findings, the authors classified interventions as ‘anthocyanin-rich’ based on the dose of anthocyanins that were consumed in a study, rather than the food name alone. Studies were included when the intervention (i) provided a quantified anthocyanin dose (e.g., mg·day^−1^ of anthocyanins or anthocyanidins), and/or (ii) used an extract or ingredient standardised to anthocyanins (i.e., anthocyanins were the specified marker for the product). Where available and reported, compositional information (e.g., HPLC/LC–MS profiling or certificate of analysis) was extracted to verify anthocyanin content and to describe the dominant anthocyanidin class in the supplement. Because whole foods, juices, concentrates and extracts contain multiple polyphenol subclasses, it cannot be excluded that the observed effects were solely due to the anthocyanins unless the administered product was anthocyanin-standardised or provided a high quantified anthocyanin dose.

## 4. Effects of Anthocyanin-Rich Sources on Exercise-Induced Substrate Oxidation

### 4.1. Blackcurrant

Table 1 contains the blackcurrant studies that are covered in the present narrative review. All blackcurrant studies used capsules that contained anthocyanins and natural sugars. The first observations for an effect on exercise-induced substrate oxidation were in 2015 with a blackcurrant extract. Cook et al. [22] had endurance-trained male cyclists undertake 10 min of cycling at 45, 55 and 65% $\dot{V}$O_2max_ following 7-day intake of 105 mg of anthocyanins in New Zealand blackcurrant (NZBC) extract. Cycling-induced fat oxidation demonstrated a trend for a change at 45% $\dot{V}$O_2max_ (placebo: 0.26 ± 0.10 vs. NZBC extract: 0.29 ± 0.09 g·min^−1^, *p* = 0.077) and 55% $\dot{V}$O_2max_ (placebo: 0.33 ± 0.14 vs. NZBC extract: 0.38 ± 0.09 g·min^−1^, *p* = 0.102) to be 15 and 13% higher than placebo, with no change in carbohydrate oxidation. At 65% $\dot{V}$O_2max_, cycling-induced fat oxidation was 27% higher with blackcurrant extract (placebo: 0.37 ± 0.15 vs. NZBC extract: 0.44 ± 0.12 g·min^−1^, *p* = 0.044) with the carbohydrate oxidation showing a trend (*p* = 0.06) to be 8% lower (Placebo: 2.36 ± 0.54 vs. NZBC extract: 2.23 ± 0.48 g·min^−1^). The same research group then followed with a dose–response study examining 105, 210, and 315 mg of anthocyanins in NZBC extract (300, 600 and 900 mg extract capsules, respectively) for 7 days on substrate oxidation during 120 min cycling at the moderate intensity of 65% $\dot{V}$O_2max_ in endurance-trained male cyclists [23]. The NZBC extract changed cycling-induced fat oxidation in a dose–response manner, with an ~18% increase for 105 mg·day^−1^, 21.5% for 210 mg·day^−1^ and 24.1% for 315 mg·day^−1^ of anthocyanins with carbohydrate oxidation also showing dose–response changes with lower values than in the placebo condition.

Now for endurance-trained female cyclists, undergoing cycling for the same duration (i.e., 120 min) and intensity (i.e., 65% $\dot{V}$O_2max_) and dosing with 210 mg·day^−1^ NZBC anthocyanins (600 mg·day^−1^ NZBC extract capsules) for 7 days, Strauss et al. [24] replicated the earlier finding in males [23] with a 27% increase in fat oxidation (placebo: 0.63 ± 0.20 vs. NZBC extract: 0.74 ± 0.13 g·min^−1^, *p* = 0.047) and a trend for a 12% decrease in carbohydrate oxidation (placebo: 1.30 ± 0.36 vs. NZBC: 1.15 ± 0.26 g·min^−1^, *p* = 0.064). In another study in females (*n* = 12), Willems et al. [25] observed a treadmill walking-induced increase in fat oxidation of 25% and a reduction in carbohydrate oxidation of 10.8% with the 7-day intake of blackcurrant extract (210 mg·day^−1^ NZBC anthocyanins).

These initial blackcurrant studies with observations on exercise-induced substrate oxidation effects were undertaken at universities within the United Kingdom. While not reported within the studies, it can be assumed that the majority of the participants in Cook et al. [22,23] and Strauss et al. [24] were of European descent. A recent editorial in the Journal of the American Medical Association emphasises that race and ethnicity are social constructs, and transparent reporting was recommended for how data was collected, why it was assessed and how categories were defined [26]. This is not consistently done in the sports nutrition literature. Willems et al. [27] examined fat oxidation during 30 min of moderate-intensity treadmill walking (5 METs) in healthy men from Southeast Asia (study in Thailand) following 7 days of supplementation with NZBC extract (210 mg of anthocyanins per day), and no effect was observed on the exercise-induced fat and carbohydrate oxidation (e.g., fat oxidation, placebo: 0.18 ± 0.13 vs. NZBC extract: 0.19 ± 0.10 g·min^−1^). In a matching method for exercise modality and intensity, but following 14-day intake of 210 mg·day^−1^ NZBC anthocyanins in healthy Southeast Asian females (study in Thailand), Willems et al. [28] also observed no effect for the walking-induced substrate oxidation (e.g., fat oxidation, placebo: 0.175 ± 0.065 vs. NZBC extract: 0.173 ± 0.073 g·min^−1^). However, studies on the response variability to supplementation should be based upon sound biological or methodological rationale, rather than just ethnicity alone, and should consider the habitual diet, training status of the participants, study design, and genetic phenotype. Factors that contribute to the individual responses to nutritional interventions are currently an important area of interest within sports and exercise nutrition. For example, a role for genetics is apparent for the inter-individual response of caffeine on time-trial performance with different expressions of genetic polymorphisms [29]. As far as we know, there is no research on the potential role of genetic polymorphisms to explain exercise-induced substrate oxidation effects due to intake of blackcurrant anthocyanins.

Applied studies in extreme altitude and hot environments have shown an inconsistent effect of NZBC anthocyanins on exercise-induced substrate oxidation. For example, Willems et al. [30] had trained cyclists undertake 10 min of cycling at 45, 55 and 65% $\dot{V}$O_2max_ following 7-day intake of 600 mg·day^−1^ NZBC extract (210 mg·day^−1^ anthocyanins) in normobaric hypoxia (~2500 m, 15% O_2_) and did not show any changes in substrate oxidation. However, during 60 min of treadmill running at 65% $\dot{V}$O_2max_ in hot ambient temperature (34 °C, 45% relative humidity), Hiles et al. [31] observed a ~30% increase in running-induced fat oxidation (placebo: 0.53 ± 0.05 vs. NZBC extract: 0.63 ± 0.06 g·min^−1^, *p* = 0.008) and a concomitant decrease in carbohydrate oxidation (placebo: 2.24 ± 0.05 vs. NZBC extract: 2.00 ± 0.07 g·min^−1^, *p* = 0.0136) from the 7-day intake of 210 mg·day^−1^ NZBC anthocyanins. Hot and high-altitude environments have unique demands as they both increase carbohydrate oxidation during exercise [32,33]. More studies are required to examine blackcurrant effects on exercise-induced substrate oxidation in hot and high-altitude environments.

For all these blackcurrant extract studies [22,23,24,25,26,27,28,30,31], the dosing duration was 7- or 14 days. This was consistent with other exercise studies dosing with anthocyanins (i.e., 8-day: Bowtell et al. [34], 4-day: Connolly et al. [35], and 6-day: Howatson et al. [36]). Therefore, the dosing strategy over days (i.e., timing, dose and intake duration) of blackcurrant anthocyanins on exercise-induced substrate oxidation may be important for eliciting responses. Following an acute intake of NZBC extract anthocyanins, Pastellidou et al. [37] reported no change in fat oxidation at the lactate threshold during an incremental running protocol in recreationally active males (placebo: 0.59 ± 0.26 vs. NZBC extract: 0.56 ± 0.44 g·min^−1^). Montanari et al. [38] examined acute and chronic intake responses during 10 min cycling at 65% $\dot{V}$O_2max_ comparing 105 mg and 210 mg NZBC extract anthocyanins for acute, 4- and 7-day intake in trained male cyclists, and no changes in exercise-induced substrate oxidation were observed. Şahin et al. [39] examined the effects of 210 mg·day^−1^ NZBC extract anthocyanins during moderate-intensity treadmill walking (4–5 METs), and this was the first study that examined the responses following 7 and 14 days of intake. The authors demonstrated increases in fat oxidation at 7 and 14 days, but carbohydrate oxidation was only changed after 14 days of intake. Lastly, in the same cohort, Şahin et al. [40] compared 14-day intake of 210 mg·day^−1^ NZBC anthocyanins, when dosing was taken daily or intermittently (i.e., every-other-day) to examine the effects during moderate-intensity treadmill walking (4–5 METs). Interestingly, only the continuous dosing pattern demonstrated a 17 ± 26% change in walking-induced fat oxidation, with a trend (*p* = 0.069) for 8 ± 13% lower carbohydrate oxidation. Interestingly, Wangdi et al. [41] demonstrated that Montmorency cherry concentrate improved 15 km cycling time-trial performance only when consumed ~90 min pre-exercise, with no improvement at 30 min or 150 min. The timing effect at ~90 min pre-exercise is consistent with the mechanistic data in Wangdi et al. [41] showing higher pre-exercise plasma phenolic metabolites (e.g., vanillic and ferulic acids) in the 30 and 90 min conditions compared with the un-supplemented and 150 min conditions, implying that the ergogenic effects for performance are more likely when the exercise coincides with elevated circulating phenolic metabolites. In Wangdi et al. [41], there were no measurements of substrate oxidation.

Of interest in the study from Şahin et al. [40] was the correlation analysis of responses with anthropometry and body composition parameters. Şahin et al. [40] demonstrated significant correlations between body mass index (r^2^ = 0.3968, *p* = 0.009) and body fat percentage (r^2^ = 0.5662, *p* < 0.001) and the absolute change in walking-induced fat oxidation from NZBC extract anthocyanins. These observations have been further examined in the first study to directly compare responses between males and females to NZBC anthocyanins by Cook et al. [42]. In Cook et al. [42], 11 males and 11 females completed 60 min of treadmill exercise at 50% $\dot{V}$O_2max_ following 7 days of 210 mg·day^−1^ NZBC extract anthocyanins and observed an increase in exercise-induced fat and a decrease in carbohydrate oxidation for the cohort (*n* = 22), with no differences in responses between males and females. Cook et al. [42] also correlated the magnitude of exercise-induced substrate responses with body fat percentage, fat-free mass and fat mass, demonstrating that only females had a significant correlation with the change in fat oxidation and body fat percentage (r = 0.691, *p* = 0.019), providing some evidence for predicting those who could respond.

A secondary analysis of seven cohort and three case studies containing data from 46 females and 71 males by Willems and Cook [43] was also able to identify that exercise-induced substrate oxidation responses from NZBC extract anthocyanins were significantly correlated to the exercise-induced respiratory exchange ratio in the placebo/control condition. Hence, those who had a higher respiratory exchange ratio (i.e., more carbohydrate oxidation), while on the control or placebo, had the greatest capacity to respond to NZBC extract anthocyanins. The analysis also demonstrated no evidence for a difference between males and females for the change in exercise-induced substrate oxidation from NZBC extract anthocyanins. In turn, the secondary analysis provides insight into factors that could identify responders or non-responders. In addition, the data in Willems and Cook [43] may suggest that some individuals can respond with enhanced exercise-induced carbohydrate oxidation and lower fat oxidation. Such observations have important implications for the recommendation of anthocyanin-rich blackcurrant by professionals in sport and exercise nutrition. However, alternative explanations for responders/no-responders, such as differences in pre-exercising muscle glycogen, which is associated with lower respiratory exchange ratio with lower muscle glycogen levels [44], cannot be entirely ruled out. In addition, most studies had morning testing using standardised pre-testing controls and allowing a light breakfast.

A further secondary analysis from a recent systematic review and meta-analysis examining the effects of blackcurrant anthocyanin supplementation on exercise-induced substrate oxidation reported that 8 of the 15 included studies demonstrated increased fat oxidation (+0.042 g·min^−1^; 95% CI: 0.017 to 0.068 g·min^−1^; *p* < 0.001) and reduced carbohydrate oxidation (−0.099 g·min^−1^; 95% CI: −0.176 to −0.022 g·min^−1^; *p* = 0.012) [12]. This systematic review and meta-analysis considered all available cohort studies on the topic without performing subgroup analyses on the influence of dose or supplementation duration, and it also incorporated studies conducted in environmentally challenging conditions (i.e., hot and high altitude).

Case studies provide a practical illustration of application. As far as we know, four case studies have examined NZBC extract anthocyanins with two reporting increased exercise-induced fat oxidation and reduced carbohydrate oxidation in males [45,46], while one reported changes for the male but not the female participant in hot conditions [47], and one reporting lower fat oxidation in a female (Southeast Asian) endurance athlete. These studies employed prolonged aerobic exercise protocols (4 h cycling, male Ironman athlete [45]; 2 h running, male ultra-endurance athletes [46]; 1 h running, Marathon des Sables athletes [47,48]) and assessed physiological and metabolic changes under controlled laboratory conditions. Collectively, the existing case studies provide preliminary evidence that well-trained ultra-endurance athletes can also experience enhanced exercise-induced fat oxidation following blackcurrant extract supplementation, despite the likelihood that such athletes already possess training-induced adaptations that favour greater fat oxidation during exercise [49].

The causes for the altered exercise-induced substrate oxidation by the intake of NZBC extract anthocyanins are not clear from these applied studies. Although it has been suggested that higher fat oxidation with lower carbohydrate oxidation could increase exercise capacity or performance from glycogen sparing, as far as we know, the evidence in support is absent from the literature. With respect to the effect of blackcurrant extract to alter exercise-induced substrate oxidation and affect endurance performance, Jones et al. [50] was the first study to provide mechanistic insights with *m.vastus lateralis* biopsies before, at 30 min and at 120 min during 120 min cycling at 65% $\dot{V}$O_2max_ in trained male cyclists following 7 days of 210 mg·day^−1^ NZBC extract anthocyanins. Following the 120 min cycling, the participants completed an exercise capacity test, cycling at 150% of lactate threshold until volitional exhaustion. Jones et al. [50] observed a 24% increase in fat oxidation (*p* = 0.025), and there may have been a mean 6% lower carbohydrate oxidation (*p* = 0.097). In addition, there may have been enhanced exercise capacity in the blackcurrant extract condition (NZBC extract: 217 ± 104, vs. PLA: 156 ± 83 s, *p* = 0.081). What was unexpected is that during the 120 min cycling, rate of muscle glycogen utilisation was greater following NZBC extract anthocyanins (NZBC: 3.19 ± 1.21 vs. PLA: 1.80 ± 0.70 mmol·kg^−1^·min^−1^), but at the end of the exercise, muscle glycogen levels were the same [50]. This was explained by a higher net breakdown of muscle glycogen in the NZBC condition (NZBC: 383 ± 145 vs. PLA: 216 ± 84 mmol∙kg^−1^ dry weight), and higher pre-exercise muscle glycogen concentrations (NZBC: 493 ± 85 vs. PLA: 355 ± 65 mmol∙kg^−1^ dry weight, *p* = 0.007). The muscle glycogen observations were also paired with the intake of NZBC extract anthocyanins to have greater biopsy-confirmed utilisation of intramuscular triglyceride in type I muscle fibres. The study by Jones et al. [50] has provided insight for a mechanism to explain the multiple findings of increased exercise-induced fat oxidation with NZBC anthocyanin supplementation. Alterations in lipolysis were also shown by Strauss et al. [24] in female endurance athletes, with 49% higher non-esterified fatty acids (NEFA) (*p* = 0.034) and 27% higher glycerol (*p* = 0.051) at rest before exercise with intake of NZBC extract; however, this needs further investigation as it was not repeated in Jones et al. [50] with male endurance athletes.

In the systematic review and meta-analysis by Cook et al. [12], the anticipated change in exercise-induced fat oxidation from NZBC anthocyanin supplementation was +0.042 g·min^−1^ (95% CI: 0.017, 0.068 g·min^−1^). Extrapolated to 60 min of steady-state moderate-intensity continuous exercise, this equates to an additional 2.5–4.1 g·h^−1^ of fat metabolised. In comparison, training interventions typically elicit larger adaptations, with increases in fat oxidation ranging from +0.12 to +0.22 g·min^−1^ (~13 g·h^−1^) [51]. Although the magnitude of change associated with NZBC anthocyanins is smaller, it should be interpreted within context. For example, some of the studies observing an increase in exercise-induced fat oxidation were undertaken in trained individuals, who would be expected to have enhanced fat oxidation capacity. Therefore, an additional increase in fat oxidation from NZBC anthocyanins for these individuals is important to recognise, but the implications on endurance performance are less clear. The meta-analysis by Braakhuis et al. [52] identified a small significant improvement in exercise performance from NZBC anthocyanins. However, due to the varied physiological responses from consumption (e.g., antioxidant, anti-inflammatory and altered enzyme expression), changes in exercise-induced substrate oxidation may not be the contributing factors here. Especially as exercise performance measurements are likely undertaken at intensities where exercise-induced fat oxidation is likely to be low or negligible (i.e., >65% $\dot{V}$O_2max_).

**Table 1:** Summary of blackcurrant studies on exercise-induced substrate oxidation. Confidence intervals and effect sizes were presented when available from the studies that reported a change in exercise-induced substrate oxidation. All studies used indirect calorimetry for the measurement of substrate oxidation.

Study	*n*; Participants; Design	Anthocyanin Dose; Intake Duration	Exercise Protocol; Conditions	Outcomes
Cook et al., 2015 [22]	14; trained male cyclists; randomised, cross-over, double-blind, placebo-controlled	105 mg·day^−1^; 7 days	stationary cycling; 10 min at 45%, 55% and 65% $\dot{V}$O_2max_	FATox: ↑ 27% at 65% $\dot{V}$O_2max_ (PL: 0.37 ± 0.15, NZBC extract: 0.44 ± 0.12 g·min^−1^, *p* = 0.044); trends at 45% and 55% $\dot{V}$O_2max_. CHO: ↓ ~8% trend at 65% $\dot{V}$O_2max_ (PL: 2.36 ± 0.54, NZBC: 2.23 ± 0.48 g·min^−1^, *p* = 0.06, no change at 45% and 55% $\dot{V}$O_2max_.
Cook et al., 2017 [23]	15; trained male cyclists; dose–response, randomised Latin square	105, 210, 315 mg·day^−1^; 7 days	stationary cycling; 120 min at 65% $\dot{V}$O_2max_	FATox: ↑ dose–response (~18% with 105 mg·day^−1^, 21.5% with 210 mg·day^−1^, 24.1% with 315 mg·day^−1^ anthocyanins. CHO: no change
Strauss et al., 2018 [24]	16; trained female cyclists, randomised, cross-over, double-blind, placebo-controlled	210 mg·day^−1^; 7 days	stationary cycling; 120 min at 65% $\dot{V}$O_2max_	FATox: ↑ 27% (PL: 0.63 ± 0.20, NZBC extract: 0.74 ± 0.13 g·min^−1^, *p* = 0.047. CHO: ↓ ~12% trend at (PL: 2.36 ± 0.54, NZBC: 2.23 ± 0.48 g·min^−1^, *p* = 0.064.
Willems et al., 2022 [25]	12; recreationally active females, randomised, cross-over, double-blind, placebo-controlled	210 mg·day^−1^; 7 days	treadmill walking; 30 min at 5-METs	FATox: ↑ 25%. (PL: 95%CI (0.19, 0.30 g·min^−1^), NZBC extract: 95%CI (0.24, 0.34 g·min^−1^), d = 0.59). CHO: ↓ 10.8%. (PL: 95%CI (0.51, 0.91 g·min^−1^), NZBC: 95%CI (0.45, 0.69 g·min^−1^), d = −0.56).
Willems et al., 2018 [27]	17; recreationally active Southeast Asian males, randomised, cross-over, double-blind, placebo-controlled	210 mg·day^−1^; 7 days	treadmill walking; 30 min at 5-METs	FATox: no change. CHO: no change.
Willems et al., 2019 [30]	11; trained male cyclists, randomised, cross-over, double-blind, placebo-controlled	210 mg·day^−1^; 7 days	stationary cycling 10 min at 45, 55, 65% $\dot{V}$O_2max_ in normobaric hypoxia (~2500 m; 15% O_2_)	FATox: no change. CHO: no change.
Hiles et al., 2020 [31]	18 (12 male); recreationally active, randomised, cross-over, double-blind, placebo-controlled	210 mg·day^−1^; 7 days	running 60 min at 65% $\dot{V}$O_2max_ in the heat (34 °C; 45% relative humidity)	FATox: ↑ ~30% (PL: 0.53 ± 0.05, NZBC extract: 0.63 ± 0.06 g·min^−1^; *p* = 0.008). CHO-ox: ↓ (PL: 2.24 ± 0.05, NZBC extract: 2.00 ± 0.07 g·min^−1^; *p* = 0.014).
Pastellidou et al., 2021 [37]	15; recreationally active males, randomised, cross-over, double-blind, placebo-controlled	105 mg·day^−1^; 8 days	incremental treadmill running; FATox assessed at lactate threshold	FATox: no change. CHO: no change.
Montanari et al., 2020 [38]	13 (12 reported substrate observations); trained male cyclists, randomised, cross-over, double-blind, placebo-controlled, repeated testing over months	105 and 210 mg·day^−1^; acute, 4- and 7 days	stationary cycling 10 min at 65% $\dot{V}$O_2max_	FATox: no change. CHO: no change.
Şahin et al., 2021 [39]	16; recreationally active males, randomised, cross-over, controlled	210 mg·day^−1^; 7- and 14 days	treadmill walking; 30 min at 4- (*n* = 3) or 5-METs	FATox: ↑ 11%, 7 days (control: 0.36 ± 0.12 (95%CI [0.30, 0.42 g·min^−1^]), NZBC extract: 0.39 ± 0.13 g·min^−1^ (95%CI [0.32, 0.46 g·min^−1^]) *p* < 0.05 ↑ 17%, 14 days (control: 0.36 ± 0.12 (95%CI [0.30, 0.42 g·min^−1^]), NZBC extract: 0.41 ± 0.13 g·min^−1^ (95%CI [0.34, 0.48 g·min^−1^]) *p* < 0.05. CHO: no change, 7 days; ↓ 9%, 14 days (control: 0.95 ± 0.40 (95%CI [0.74, 1.17 g·min^−1^]), NZBC extract: 0.86 ± 0.33 g·min^−1^ (95%CI [0.68, 1.03 g·min^−1^]), *p* < 0.05).
Şahin et al., 2022 et al., [40]	16; recreationally active males, randomised, cross-over, controlled	210 mg·day^−1^; 14 days every day and every-other-day	treadmill walking; 30 min at 4- (*n* = 3) or 5-METs	FATox: ↑ 17%, 14 days (control: 0.36 ± 0.12 (95%CI [0.30, 0.42 g·min^−1^]), NZBC extract: 0.41 ± 0.13 g·min^−1^ (95%CI [0.34, 0.48 g·min^−1^]), d = 0.40, *p* < 0.05. No change: 14 days every-other-day. CHO: ↓ 9%, 14 days (control: 0.95 ± 0.40 (95%CI [0.74, 1.17 g·min^−1^]), NZBC extract: 0.86 ± 0.33 g·min^−1^) (95%CI [0.68, 1.03 g·min^−1^]). No change: 14 days every-other-day.
Cook et al., 2025 [42]	22 (11 males); recreationally active, randomised, cross-over, double-blind, placebo-controlled	210 mg·day^−1^; 7 days	treadmill walking or running; 60 min at 50% $\dot{V}$O_2max_	FATox: ↑ (PL: 0.21 ± 0.12, NZBC extract: 0.27 ± 0.11 g·min^−1^, d = 0.859, *p* < 0.001). CHO: ↓ (PL: 1.43 ± 0.49, NZBC extract: 1.32 ± 0.44 g·min^−1^, d = −0.762, *p* = 0.002).
Willems et al., 2024 [45]	case study; male Ironman athlete; randomised, single blind, placebo-controlled	acute intake: 420 mg 2 h before	4 h indoor stationary cycling at 165 Watts	FATox (trend): ↑ 13% (placebo: 0.50 ± 0.06 (95%CI [0.45, 0.55 g·min^−1^]), NZBC extract: 0.56 ± 0.05 g·min^−1^ (95%CI [0.52, 0.61 g·min^−1^]), *p* = 0.096). CHO-ox: ↓ 11% (placebo: 2.04 ± 0.17 g·min^−1^ (95%CI [1.90, 2.18 g·min^−1^]), NZBC extract: 1.80 ± 0.13 g·min^−1^ (95%CI [1.69, 1.91 g·min^−1^]), *p* = 0.025).
Willems and Briggs 2022 [46]	case study; male ultra-endurance runner	210 mg·day^−1^ for 7 days	2 h treadmill running at 10.5 km·h^−1^ (~58% $\dot{V}$O_2max_) at 26 °C and ~70% RH)	FATox: ↑ 23% (control: 0.39 ± 0.08 (95%CI [0.32, 0.47 g·min^−1^]), NZBC extract: 0.48 ± 0.12 g·min^−1^ (95%CI [0.38, 0.59 g·min^−1^]), *p* < 0.002). CHO-ox: ↓ 11% (Control: 1.94 ± 0.12 g·min^−1^ (95%CI [1.85, 2.04 g·min^−1^]), NZBC extract: 1.73 ± 0.21 g·min^−1^, (95%CI [1.55, 1.91 g·min^−1^]), *p* = 0.01.
Willems et al., 2024 [47]	two case studies; female and male Marathon des Sables athletes, non-acclimatised	210 mg·day^−1^ for 7 days	1 h treadmill running at 50% $\dot{V}$O_2max_ (34 °C and 30% relative humidity).	Female: no change. Male FATox: ↑ 21% (control: 0.84 ± 0.11, NZBC extract: 1.02 ± 0.08 g·min^−1^, *p* = 0.009). CHO-ox: ↓ 31% (control: 1.07 ± 0.28, NZBC extract: 0.71 ± 0.12 g·min^−1^, *p* = 0.05)
Willems et al., 2026 [48]	case study; female endurance athlete (Southeast Asian)	420 mg·day^−1^ for 7 days	1 h treadmill running at 50% $\dot{V}$O_2max_ (24 °C and 42% relative humidity).	FATox: ↓ 7% [placebo: 95%CI (0.39, 0.48 g·min^−1^)], NZBC extract: 95%CI (0.36, 0.45 g·min^−1^)], *p* < 0.01. CHO-ox: ↑ 43% [placebo: 95%CI (0.33, 0.51 g·min^−1^), NZBC extract: 95%CI (0.50, 0.70 g·min^−1^), *p* < 0.01)
Jones et al., 2026 [50]	10; trained male cyclists; randomised, cross-over, double-blind, placebo-controlled	210 mg·day^−1^; 7 days	stationary cycling 120 min at 65% $\dot{V}$O_2max_ + capacity test	FATox: ↑ 24% (*p* = 0.026). CHO: ↓ 6% trend (*p* = 0.097).

CHO, carbohydrate oxidation; FATox, fat oxidation; MET, metabolic equivalent of task; ↑ increase; ↓ decrease. “trend” indicates reported *p* values between 0.05 and 0.10.

### 4.2. Blueberry and Cherry

Table 2 contains the blueberry, tart cherry, blackberry and elderberry studies that are covered in this narrative review. Blackcurrant has received the most research interest for the effects on exercise metabolism. However, other berries and fruits that are high in anthocyanin content have also been examined and this is important because berries possess distinct anthocyanin profiles [10]. However, it cannot be excluded that in addition to the anthocyanins, other bioactive compounds may have contributed to the observations on exercise-induced substrate oxidation with blueberry, tart cherry, blackberry and elderberry.

In a three-arm, non-cross-over trial involving a placebo, tart cherry concentrate (320 mg anthocyanins; *n* = 14), and blueberry concentrate (387 mg anthocyanins; *n* = 15), Sinclair et al. [53] observed no change in substrate oxidation during a 6 min treadmill walk at 4.5 km·h^−1^ following 20 days of supplementation in adults. A strength of the study by Sinclair et al. [53] was the comparison of the physiological outcomes between two high anthocyanin containing foods, allowing insight (even with the no response) into whether specific anthocyanin profiles or foods are important for observed responses. Using a similarly lengthy supplementation period, Pilolla et al. [54] administered 12.5 g whole freeze-dried blueberry powder in 125 mL of water twice daily (providing 375 mg·day^−1^ anthocyanins) for two weeks during a controlled low-polyphenol diet and observed a 43% increase in exercise-induced fat oxidation during 40 min of cycling at 65% $\dot{V}$O_peak_ after a 12 h overnight fast. To the author’s knowledge, these are presently the only studies examining if blueberry can influence exercise-induced substrate oxidation, and it warrants further investigation. It needs to be recognised that elements of the experimental design such as exercise testing in a fasted state may affect, in itself, the metabolic responses (e.g., [55]), and it cannot be excluded to affect the anthocyanin-induced responses as well.

Desai et al. [56] investigated whether 20 days of Montmorency tart cherry juice supplementation (540 mg·day^−1^ anthocyanins) could alter exercise-induced substrate oxidation during exercise. After a minimum of a 10 h overnight fast, participants completed cycling trials at an individualised intensity to elicit their maximal fat oxidation rate (~45% $\dot{V}$O_peak_) pre-, mid- (day-10), and post-supplementation (day-21). Compared with placebo, the Montmorency cherry supplement did not alter maximal fat oxidation rates or the exercise intensity at which this occurred across any of the testing visits. Similarly, Gao et al. [57] examined the effects of short-term tart cherry juice supplementation on substrate oxidation during steady-state cycling in a double-blind, cross-over design. Recreational cyclists consumed 300 mL·day^−1^ of tart cherry juice (providing ~2760 mg·day^−1^ anthocyanins/~9.2 mg·mL^−1^) or a sports drink (0 mg anthocyanins) for four days prior to the exercise. Following a 10 h overnight fast, participants consumed a calorie matched (providing 1 g·kg^−1^ carbohydrate) sports drink or tart cherry (~4625 mg anthocyanins) drink 45 min before completing 90 min of cycling at 50% of maximal workload. The tart cherry juice did not alter carbohydrate oxidation and fat oxidation during the 90 min submaximal cycling trial compared with the sports drink condition. The absence of effect was despite a substantially higher anthocyanin dose than in many blackcurrant studies.

**Table 2:** Summary of the anthocyanin-rich blueberry, tart cherry, blackberry and elderberry studies on exercise-induced substrate oxidation. All studies used indirect calorimetry for the measurement of substrate oxidation.

Study	*n*; Participants; Design	Food; Anthocyanin Dose; Intake Duration	Exercise Protocol; Conditions	Outcomes
Sinclair et al., 2022 [53]	15 blueberry (9 male), 14 cherry (7 male), 15 placebo (8 male), non-obese healthy adults, 3-arm trial; randomised, single-blind, placebo-controlled	tart cherry; 640 mg·day^−1^, 20 days; blueberry; 774 mg·day^−1^, 20 days	6 min treadmill walk at 4.5 km·h^−1^.	FATox: no change. CHO: no change.
Pilolla et al., 2023 [54]	11; aerobically trained males; non-randomised, cross-over	blueberry powder, 375 mg·day^−1^; 2 weeks alongside low-polyphenol diet	stationary cycling; 120 min at 65% $\dot{V}$O_2peak_	FATox 20 min: ↑ 19.7% (control: 0.38 ± 0.22 g·min^−1^, blueberry 0.49 ± 0.23 g·min^−1^, *p* = 0.049). FATox 30 min: ↑ 43.2% (control: 0.35 ± 0.20 g·min^−1^, blueberry 0.49 ± 0.26 g·min^−1^, *p* = 0.010). FATox 40 min: ↑ 31.1% (control: 0.38 ± 0.23 g·min^−1^, blueberry 0.50 ± 0.25 g·min^−1^, *p* = 0.012). CHO 20 min: ↓ 10.1% (control: 1.84 ± 0.96 g·min^−1^, blueberry 1.65 ± 0.83 g·min^−1^, *p* = 0.024). CHO 30 min: ↓ 19.2% (control: 1.89 ± 0.96 g·min^−1^, blueberry 1.54 ± 0.81 g·min^−1^, *p* = 0.014). CHO 40 min: ↓ 14.8% (control: 1.78 ± 0.93 g·min^−1^, blueberry 1.52 ± 0.79 g·min^−1^, *p* = 0.045).
Desai et al., 2018 [56]	11 (7 males); trained female cyclists; randomised cross-over, single-blind, placebo-controlled and counterbalanced	tart cherry juice; 540 mg·day^−1^; 20 days	stationary cycling; 60 min at FATmax (~45% $\dot{V}$O_2peak_)	FATox: no change CHO: not reported
Gao et al., 2024 [57]	12 (8 males); recreational cyclists; randomised cross-over, double-blind, placebo-controlled and counterbalanced	tart cherry juice; ~2760 mg·day^−1^; 4 days + pre-exercise ~4625 mg anthocyanins	stationary cycling; 90 min at 50% maximal workload	FATox: no change. CHO: no change.
Solverson et al., 2018 [58]	27; overweight/obese males; randomised cross-over, placebo-controlled	whole blackberries; ~361 mg·day^−1^; 7 days	treadmill walking; 30 min at 3 mi·h^−1^ (whole-room calorimeter)	FATox: ↑ total oxidised (gelatine: 8.32 g, 95%CI [7.06–9.59 g], blackberry: 9.35 g, 95%CI [8.08–10.62] (*p* = 0.004) CHO: ↓ total oxidised (gelatine: 25.71 g, 95%CI [23.07, 28.35 g], blackberry: 23.46 g, 95%CI [20.82–26.10], *p* = 0.050).
Teets et al., 2024 [59]	18 (3 male); overweight/obese; randomised, cross-over, placebo-controlled	elderberry juice; 755 mg·day^−1^; 7 days	treadmill walking; 30 min at 3 mi·h^−1^	FATox: ↑ trend total oxidised (PL: 4.32 ± 0.35 g, elderberry: 4.99 ± 0.34 g, *p* = 0.071). CHO: ↓ trend total oxidised (PL: 22.0 ± 1.2, elderberry: 20.6 ± 0.90 g; *p* = 0.055).

CHO, carbohydrate oxidation; FATox, fat oxidation; ↑ increase; ↓ decrease. “trend” indicates reported *p* values between 0.05 and 0.10.

### 4.3. Blackberry and Elderberry

The effects of blackberry on exercise-induced substrate oxidation have also been examined. Solverson et al. [58] had overweight and obese men (body mass index ≥ 25 kg/m^2^) consume an investigator controlled high-fat diet (40% fat, 45% carbohydrate, and 15% protein) that was free of anthocyanins except for those supplied through the blackberry intervention. Participants consumed either 600 g of whole blackberries per day (~361 mg anthocyanins) or an energy-matched flavoured gelatin for one week. As part of a 24 h stay within an indirect calorimetry chamber, participants completed 30 min of treadmill walking at 3 mi·h^−1^. During the treadmill walking, total fat oxidation increased by 12% in the blackberry condition compared with gelatin (gelatin: 8.32 g, 95% CI [7.06, 9.59] vs. blackberry: 9.35 g, 95% CI [8.08, 10.62], *p* = 0.004). Carbohydrate oxidation was also 10% lower with blackberry consumption (gelatin: 25.71 g, 95% CI [23.07, 28.35] vs. blackberry: 23.46 g, 95% CI [20.82, 26.10], *p* = 0.050). Using a diet with an identical macronutrient profile and the same week-long duration, Teets et al. [59] had overweight and obese participants (men: *n* = 3 and women: *n* = 15) supplemented with 355 g of elderberry juice per day (7 days, 755 mg·day^−1^ of anthocyanins). The walking-induced fat oxidation with elderberry tended to be higher (elderberry: 4.99 ± 0.33 vs. placebo: 4.32 ± 0.35 g, *p* = 0.071), with carbohydrate oxidation tending to be lower (elderberry: 20.6 ± 0.9 vs. placebo: 22.0 ± 1.2 g, *p* = 0.055). The respiratory quotient was lower with elderberry (elderberry: 0.891 ± 0.006 vs. placebo: 0.905 ± 0.007, *p* = 0.038) suggesting an increased exercise-induced fat oxidation. Both studies used low-speed treadmill walking; however, taken together, these studies provide observations that anthocyanins can enhance exercise-induced fat oxidation in people who are overweight or have obesity, which could have clinical or health implications. Studies on exercise-induced substrate oxidation and potential for weight management with intake of blackcurrant are currently absent from the literature. However, it needs to be noted that although the % changes sometimes suggest a large effect on exercise-induced fat oxidation by an anthocyanin-rich source, it is not clear whether the resulting absolute changes have meaningful application.

## 5. Methodological Considerations

An important consideration for the interpretation of the study observations on the exercise-induced metabolic effects of anthocyanin is whether dietary control was part of the methodology. In a recent meta-analysis demonstrating that blackcurrant increases exercise-induced fat oxidation [12], none of the included studies used a low-polyphenol washout diet before laboratory assessments. However, Pilolla et al. [54] observed with blueberry (whole freeze-dried powder) supplementation a 43% increase in fat oxidation, a percentage increase that was substantially higher than any studies supplementing with blackcurrant, but the 43% was obtained after a two-week low-polyphenol-controlled diet. To have strict dietary control in studies will limit the ecological validity of the findings of a study. In addition, anthocyanin metabolites are likely to act more effectively in combination than when in isolation, demonstrating synergistic effects [60,61]. While a polyphenol washout diet can isolate an effect of supplementation and confirm that the anthocyanin supplement is causing the observed response, for athletes and practitioners it is important to know that the changes in exercise-induced substrate oxidation can be observed on top of the habitual diet to have confidence in the efficacy. However, the absence of strict dietary control for a few days before testing may contribute to the variation in the anthocyanin-induced effects on substrate oxidation during exercise.

Except for one study by Solverson et al. [58], which employed a whole-room calorimeter, all studies included in this review derived substrate oxidation from indirect calorimetry using respiratory gas analysis. Substrate oxidation rates were calculated using stoichiometric equations with $\dot{V}$O_2_ and $\dot{V}$CO_2_ values [62,63]. Firstly, this assumes that gluconeogenesis, lipogenesis, and ketogenesis are not occurring and influencing the respiratory exchange ratio. Secondly, it ignores protein metabolism during exercise and assumes this is consistent or negligible. The contribution of protein is much smaller than carbohydrate or fat during prolonged exercise and is likely ≤5% [64], but possibly up to ~20% [65].

The studies that were covered in this narrative review had different delivery formats of anthocyanin supplementation. Many of the blackcurrant studies used spray-dried and encapsulated blackcurrant extract, whereas some studies supplemented with anthocyanins from the food such as blueberries. Anthocyanins present within the original food may provide protective effects of the stomach environment, allowing them to reach the intestinal environment [66] and be broken down by the gut bacteria. At present, there is no evidence to confirm whether the delivery format of the anthocyanin consumption is important for effects on exercise-induced substrate oxidation.

### 5.1. Energy Expenditure Responses to Anthocyanin Supplementation

Although the focus of the present narrative review is on the effects of anthocyanins on substrate oxidation during exercise, consideration of overall energy expenditure is relevant, as fat and carbohydrate oxidation directly contribute to energy expenditure. Several of the studies that were included in this review did not report on energy expenditure. This includes investigations involving blackcurrant supplementation [30,31,42,45,46,47,48] and one blueberry study [54]. In contrast, the studies that quantified the energy expenditure did not report any significant effects, regardless of the anthocyanin source. This includes blackcurrant studies [22,24,39,40,50], research combining cherry and blueberry [53], and studies on elderberry [59] and blackberry [58].

Consistent across all the studies was that there was no change in oxygen consumption during exercise with intake of anthocyanins. It is important to recognise that fat oxidation and energy expenditure are not coupled [67], and carbohydrate oxidation will change to maintain a consistent energy expenditure. Therefore, a change in fat oxidation following anthocyanin consumption cannot be assumed to cause weight loss from fat stores during a prolonged intervention. However, the influence of anthocyanins on anti-obesity effects is instead likely linked to upregulation of the phosphoinositide 3-kinase/protein kinase B (PI3K/Akt) signalling pathway [68] rather than alterations in exercise-induced substrate oxidations.

### 5.2. Anthocyanin Profiles

Important to the interpretation of the studies presented within this review are the unique anthocyanin profiles present. Within blackcurrant, delphinidin glucosides comprise >60% of anthocyanins, for blueberry malvidin glucosides compromise ~35%, while blackberry, elderberry and cherry comprise >90% cyanidin, as calculated from the Phenol-explorer database [69]. The radical oxygen scavenging ability differs for anthocyanidins and the type of radical. For example, against the superoxide anion, the most effective is delphinidin followed by cyanidin and pelargonidin, whereas, against the hydroxyl radical, pelargonidin is the most effective [70].

Some of the foods and anthocyanin sources discussed in this review, such as blackberry and elderberry, are represented by a relatively small number of studies, and therefore their specific effects on substrate oxidation during exercise are unclear. This limited evidence highlights the need for further investigations to determine whether these lesser-studied anthocyanin-rich foods exert similar metabolic effects to those observed with more extensively researched sources, such as blackcurrant. Moreover, it is not yet clear whether unique anthocyanin profiles lead to distinct substrate oxidation responses during exercise. Clarifying these effects within one study design would provide valuable insights into whether unique anthocyanin profiles or specific berries or foods can alter exercise-induced substrate oxidation, ultimately informing more targeted sports and exercise nutrition strategies.

It is worth noting within the context of this review that some of the supplements and foods used contain multiple polyphenols (e.g., phenolic acids, flavonols, proanthocyanidins); however, the dose of anthocyanins present is likely the causal factor for the observed effects. For all the included studies, anthocyanin-rich sources were discussed where the intervention provided a defined dose of anthocyanins and where compositional profiles support that anthocyanins are present. Furthermore, analysis from the Phenol-Explorer database [69] demonstrates that anthocyanins are present in all the foods used within this review, and for all foods anthocyanins provide at minimum >40% towards the total polyphenol content (Table 3). However, the exact composition in whole blackcurrant, for example, contains multiple polyphenols and not just anthocyanins present within the capsulated extract in the studies presented in this review. For the blueberry, tart cherry, blackberry and elderberry studies, it is also likely that the supplement that was used had a different polyphenol composition than whole berries or drupes.

### 5.3. Cohort and Case Studies

In the present review are four case studies on the effects of New Zealand blackcurrant extract [45,46,47,48]. Case studies rank low in the hierarchy of scientific evidence, and that has been recognised as well for case studies with dietary supplements [71]. Another limitation needs to be noted: in the case studies by Willems and Briggs [46] and Willems et al. [47,48], a non-randomised approach was taken due to the restricted time that the athletes were available for testing in preparation for a competitive event (i.e., placebo testing first to avoid the requirement of a washout). In addition, Pilolla et al. [54] also used a non-randomised approach, making it 4 out of the 23 studies that were covered in the present review. Another potential limitation is that most of the case and cohort studies on the effects of blackcurrant were from the same research group [22,23,25,27,30,31,38,39,40,45,46,47,48], with at times the reporting of a trend for change [22,24,40,45,59], suggesting that some studies may not have had the required sample size to provide solid evidence for change in substrate oxidation by the supplement.

## 6. Future Research Directions

As previously identified, future research should determine whether specific anthocyanin-rich berries and fruits elicit distinct effects on exercise-induced substrate oxidation. Further work is also needed to identify characteristics that differentiate responders from non-responders to anthocyanin supplementation, including training status, age, intake duration and responses under extreme environmental conditions. Such issues and specific berry effects are difficult to address in separate studies as the methodological details of studies from different groups and even from the same group may have contributed to the variation in observations. Most evidence demonstrating blackcurrant-induced increases in fat oxidation has been obtained at low-to-moderate exercise intensities and primarily in untrained or moderately trained individuals [12]. Although case studies in endurance-trained athletes have reported mostly similar enhancements, these findings were still limited to moderate-intensity workloads [45,46,47]. Given that endurance-trained athletes typically achieve maximal fat oxidation at higher intensities than untrained individuals, the potential influence of blackcurrant on maximal fat oxidation and the corresponding exercise intensity remains unknown and warrants investigation.

It is common practice for athletes to consume more than one dietary supplement simultaneously. The effect of anthocyanins on exercise-induced substrate oxidation when combined with other supplements is not known. For example, caffeine is a widely consumed ergogenic aid and increases lipolysis and fat oxidation during exercise [72]; however, the combination of caffeine (and potentially other known supplements that can alter exercise-induced substrate oxidation [21]) and blackcurrant anthocyanins on metabolism is not known and needs investigating. In addition, supplementation effects during long-duration continuous exercise with dietary intake (i.e., combination of carbohydrates, caffeine and electrolytes) needs further research.

Within sports and exercise nutrition, it is common to observe responders, low responders and non-responders to the intervention. To date, there have been some investigations and analyses to determine factors that could identify those that would respond. Cook et al. [23] reported estimated daily anthocyanin intake from a food frequency questionnaire and observed no correlation to the magnitude of substrate oxidation responses in trained males during cycling (unpublished observations). Similarly, using a food frequency questionnaire, but in females and during treadmill walking, Willems et al. [25] also reported no correlation (r^2^ = 0.04, *p* = 0.56) to habitual anthocyanin intake and change in exercise-induced substrate oxidation. Therefore, current understanding does not indicate that the total habitual anthocyanin intake is able to identify those who would respond. The intrinsic exercise-induced substrate oxidation may be a factor predicting a responder with fat or carbohydrate oxidation by intake of blackcurrant anthocyanins with males or females not different [43]. Lastly, body composition may also be important, with Cook et al. [42] demonstrating that body fat percentage was correlated to the delta change in substrate oxidation from NZBC extract anthocyanins, but only in females.

**Figure 1:**
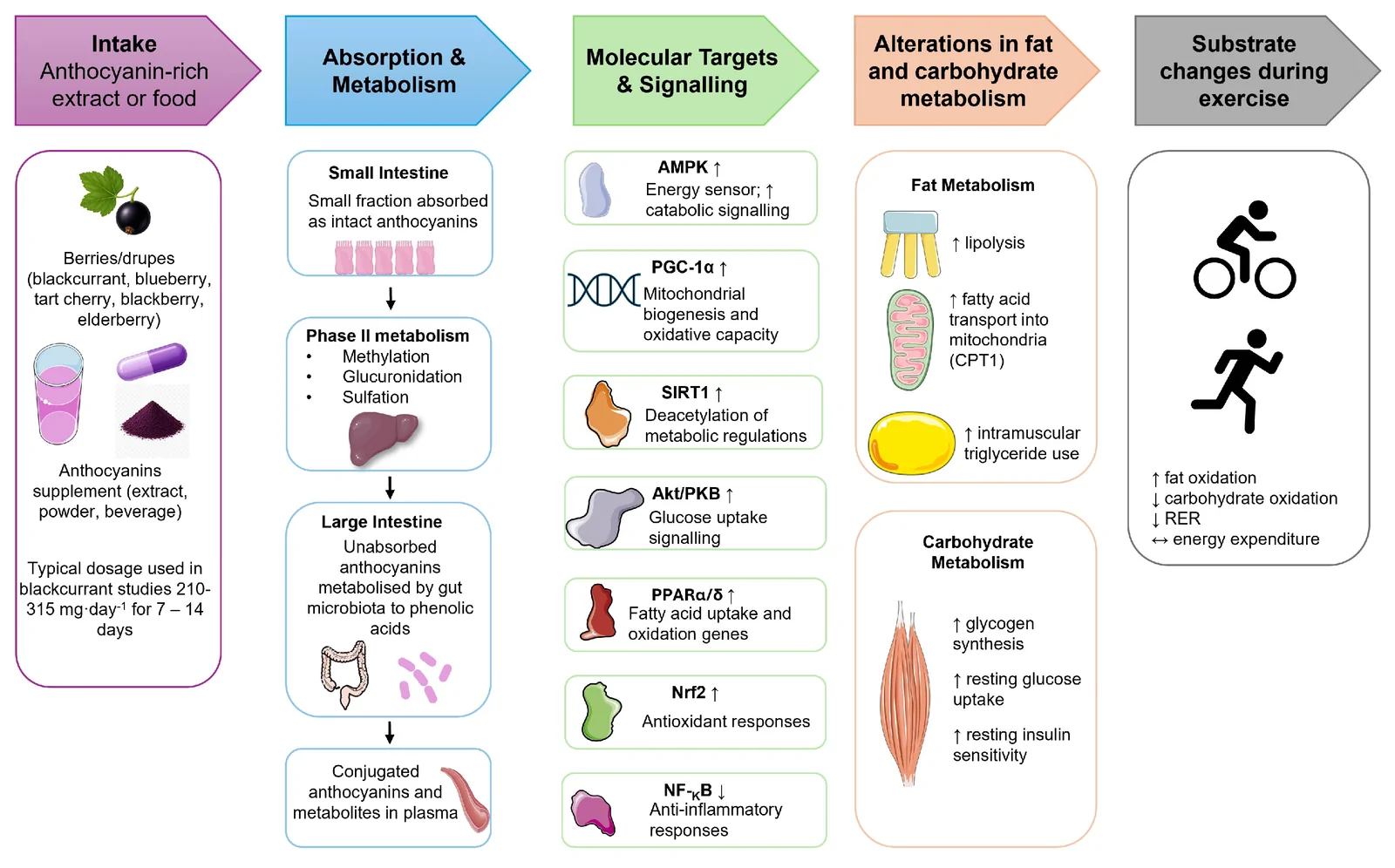
Pathways linking anthocyanin consumption, processes and potential molecular signalling targets influencing fat and carbohydrate oxidation during exercise. AMPK—adenosine monophosphate-activated protein kinase; PGC1α—peroxisome proliferator-activated receptor gamma coactivator 1-alpha; SIRT1—sirtuin 1; Akt/PKB—protein kinase B; PPARα/δ—peroxisome proliferator-activated receptor alpha/delta; Nrf2—nuclear factor erythroid 2-related factor 2; NF-_K_B—nuclear factor kappa-light-chain-enhancer of activated B cells. Images adapted from Servier Medical Art (https://smart.servier.com (accessed on 4 September 2026)), licenced under CC BY 4.0 (https://creativecommons.org/licenses/by/4.0/ (accessed on 4 September 2026)).

The research into responder versus non-responders examined potential influential factors, rather than examining the underlying mechanism. Variations in sites of metabolism, intestinal microbiota, and tissues can influence the absorption, distribution, metabolism and excretion of anthocyanins. For example, higher abundances of the gut microbiota *Bifidobacterium* spp., *Lactobacillus* spp., and *Akkermansia* may be associated with an individual being a responder. However, this relationship is not yet fully understood as the interaction between anthocyanins and the gut microbiota is bidirectional, with anthocyanins influencing the composition of the gut microbiome, and microbiota transforming unabsorbed anthocyanins into metabolites which are subsequently absorbed [73]. Lastly, genetic polymorphisms for genes that regulate phase I and II metabolism, transport proteins or enzymes in the conjugation pathways may all contribute to the complexity of inter-individual variability [74]. There is a need for combining the assessment of these factors and identifying responses to exercise-induced substrate oxidation from anthocyanin intake, which, in turn, may have health implications. As far as we know, there are no nutritionally controlled studies that aim to systematically examine predictor(s), including the role of the gut microbiota, with the simultaneous measurement of substrate oxidation, muscle biopsies for muscle glycogen and intramuscular triglycerides, plasma metabolites and relevant metabolic pathways to confirm for the responsiveness to anthocyanin-rich sources during exercise. In addition, the potential link between the anthocyanin-induced plasma metabolites and cellular effects will contribute to our understanding of the in vivo effects during exercise. Figure 1 presents a general overview of the pathways linking intake of anthocyanins and changes in exercise-induced substrate oxidation. The focus in the present narrative review was on the outcomes of the intake of anthocyanins, i.e., the exercise-induced oxidation of fat and carbohydrates during moderate-intensity exercise.

Lastly, several anthocyanin-rich berries and commercial products (e.g., mulberry, boysenberry, haskap, bilberry, elderberry, huckleberry, cranberry, silvanberry and related Rubus hybrids) have not yet been examined for their effects on substrate oxidation during exercise. Studies including the berry or product (i.e., whole polyphenol profile) and studies supplementing with specific anthocyanidins (i.e., delphinidin-3-glucose vs. cyanidin-3-glucoside) are needed to determine whether specific anthocyanins are causing effects, the complete anthocyanin profile of the food, or the complete polyphenol profile causes the responses.

## 7. Conclusions

Short-term anthocyanin supplementation from blackcurrant extract made from New Zealand-grown fruit elicits increases in fat oxidation during moderate-intensity continuous exercise, with several studies reporting ~15–30% elevations after seven days of intake. The shifts were occasionally accompanied by reduced exercise-induced carbohydrate oxidation. Alterations in exercise-induced substrate oxidation from other anthocyanin-rich supplements such as blueberry and tart cherry remain inconsistent and need further investigation. Anthocyanin-rich blackberry and elderberry have both demonstrated effects, but also more studies are needed. Anthocyanin-rich blackcurrant has shown the potential for anthocyanin-rich supplementation to have applications for sport and exercise nutrition. Future research should explore effects on maximal fat oxidation parameters, sex-specific responses, effects upon exercise performance in competitive settings, and whether the substrate oxidation responses work in cohorts of highly trained individuals and support weight loss and health in clinical populations.

## Figures and Tables

**Table 3:** Polyphenol content of anthocyanin-rich sources: blackcurrant, blueberry, tart cherry, blackberry and elderberry.

	Blackcurrant	Blueberry	Tart Cherry	Blackberry	Elderberry
total polyphenol content (mg/100 fw)	621.86	310.89	274.3	1358.66	256.69
anthocyanins (mg/100 fw, %TC)	592.22 (95.23%)	133.99 (43.1%)	171.42 (62.49%)	1316.66 (96.91%)	172.59 (67.24%)
flavanols (mg/100 fw, %)	1.17 (0.19%)	1.11 (0.36%)	15.07 (5.49%)	NR	13.87 (5.4%)
flavonols (mg/100 fw, %)	13.68 (2.2%)	38.69 (12.44%)	NR	42 (3.09%)	12.77 (4.97%)
hydroxybenzoic acids (mg/100 fw %)	1.43 (0.23%)	1.45 (0.47%)	NR	NR	50.19 (19.55%)
hydroxycinnamic acids (mg/100 fw, %)	13.36 (2.15%)	135.65 (43.64%)	87.81 (32.01%)	NR	7.27 (2.38%)

TC, total content, fw, fresh weight; NR, not reported.

## Data Availability

No new data were created or analysed in this study. Data sharing is not applicable to this article.
